# Late Preterm Antenatal Steroid Use and Infant Outcomes in a Single Center

**DOI:** 10.21203/rs.3.rs-3718685/v1

**Published:** 2023-12-11

**Authors:** Mitzi Go, Madison Wahl, Tova Kruss, Cindy McEvoy

**Affiliations:** Oregon Health & Science University

## Abstract

**Objective:**

To characterize late preterm antenatal steroids (AS) use and associated neonatal outcomes in a single academic center.

**Study Design:**

Retrospective study of 503 singleton, mother-infant dyads delivered between 34 0/7 to 36 6/7 weeks gestation between January 1, 2016 to December 31, 2020.

**Results:**

43% did not receive AS (No AS) prior to delivery. Among AS treated, 50% were sub-optimal dosing. No AS had higher preterm premature rupture of membranes and maternal diabetes. AS group had lower mean gestational age and birthweight and longer time from admission to delivery and longer NICU study. There was no difference in neonatal hypoglycemia.

**Conclusions:**

Sub-optimal AS dosing in late preterms remains high in our center. AS did not improve neonatal outcomes. Studies are needed to evaluate the impact of AS in diabetics delivering late preterm, to optimize the timing of AS dosing, and evaluate the longer term impact on late preterm infants.

## Introduction

A single course of antenatal steroids (AS) has been recommended by the Society for Maternal Fetal Medicine (SMFM) and American College of Obstetrics and Gynecology (ACOG) for women at risk of late preterm delivery (34 0/7 to 36 6/7 weeks gestation) (1, 2) after the Antenatal Late Preterm Steroids Trial (ALPS), a large, randomized, controlled trial, showed improvement in short term respiratory outcomes in late preterm infants (LPIs) (3). However, use of AS is not without potential risks, having been associated with short-term neonatal hypoglycemia in LPIs(3, 4), decreased growth (weight, length, head circumference), and higher rates of neurodevelopmental delay in exposed term infants (5–7). In a large, double-blind randomized trial in India, late preterm AS did not result in a reduction in neonatal death, stillbirth, or severe neonatal respiratory distress(8). The potential for harmful side effects speaks to a need for a sharply defined therapeutic index to maximize the potential benefits and minimize the potential harms to the infant.

Adding to this concern, a survey of practicing obstetric providers reported that the majority provided routine administration of AS for indications outside the strict inclusion and exclusion criteria from the ALPS trial (i.e. singleton pregnancies with a high probability of delivering within 7 days of randomization; excluding those expected to deliver in less than 12 hours for any reason, pregestational diabetes, known major fetal anomalies, or those who had previously received AS during pregnancy)(9). Concern for “indication creep” was also raised by McElwee, et al, when they found 229 out of 660 (35%) women in their cohort from 2016 to 2019 who received late preterm AS were deemed “inappropriate exposures” using recommended criteria(10). There is a need for better understanding of the practice patterns at various institutions and the associated neonatal outcomes associated with AS use. As a first step, this study aims to better understand the AS administration patterns during the late preterm gestation at our institution compared to SMFM recommendations, as well as to examine the short-term outcomes in exposed neonates.

## Methods

A retrospective review of electronic medical records was conducted for mother-baby dyads of singleton neonates born during the late preterm period (defined as 34 0/7 to 36 6/7 weeks gestational age (GA)) at Oregon Health & Science University (OHSU) between January 1, 2016 and December 31, 2020. The study was approved by the OHSU IRB and need for consent waived due to its retrospective nature.

Maternal baseline demographic characteristics, comorbidities, antenatal steroid use during pregnancy, tobacco use, indication for delivery, mode of delivery and date/time of delivery were collected. Maternal diabetes was further classified as pregestational – type 1 (T1DM) and type 2 (T2DM), or gestational diabetes (A1GDM and A2GDM). The definition for each type of gestational diabetes were based on current clinical practice definitions and diagnostic criteria(11). Preterm prolonged rupture of membranes (PPROM) was defined as rupture of membranes prior to 37 weeks gestation, while tobacco use was defined as any affirmative response to smoking cigarettes during pregnancy. We defined a composited outcome of “sub-optimal late AS administration” as either a latency between first dose of AS and delivery of less than 6 hours; or a latency of AS course more than 7 days prior to delivery. Neonatal data collected include GA, gender, birth weight, APGAR scores, observation or admission to the neonatal intensive care unit (NICU), need for surfactant therapy, need for respiratory support, length of hospital stay, and serum glucose measurements for the first 24 hours of life. Small for gestational age (SGA) is defined as < 10th percentile for GA, while large for gestational age (LGA) is defined as > 90th percentile for GA.

Neonatal hypoglycemia was defined as a glucose level less than 40 mg/dL at any time within the first 24 hours of life. We excluded infants with significant congenital or chromosomal anomalies and gestations that went from twins to singletons.

### Statistical analysis

We used descriptive statistics (e.g., means, medians, standard deviations, and interquartile ranges) to provide an overview of the demographic information of the birth parent and their offspring. Infants were classified into two groups based on whether or not AS were administered. We assessed associations between numerical variables (e.g., GA, mean days in the NICU) and group membership using Welch’s *t*-tests, and between categorical variables (e.g., sex, diagnosis) and group membership using Fisher’s exact tests. To model potential confounding variables, we also performed three separate multiple linear regressions to control for confounding variables that were known risk factors for low birth weight and prolonged hospital stay. First, birth weight was adjusted for the confounders of GA, smoking status in the birth parent, and diabetes. Then, mean days in the NICU and mean days of hospital stay were both adjusted for birthweight, GA, and steroid administration. Statistical significance was determined using alpha level of 0.05. All analyses were completed using R studio Version 2023.06.1 + 524.

## Results

### Mothers

**Figure 1:**
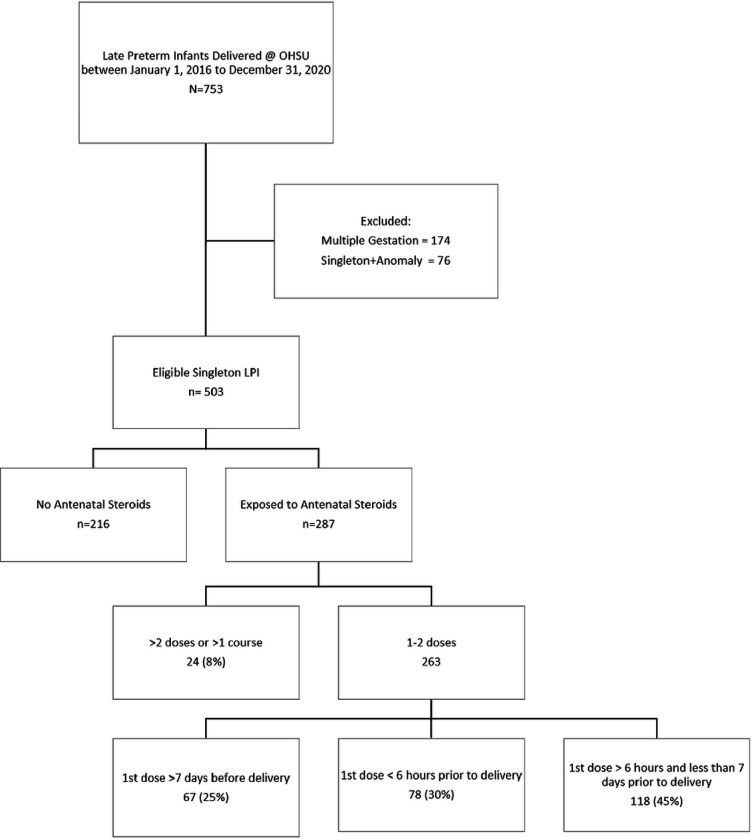
STROBE flow diagram identifying late preterm infants (LPIs) included in the analysis.

There were a total of 503 mother-infant dyads included in this analysis. Two hundred eighty-seven (57%) dyads received AS, while 216 (43%) did not receive AS. Among those who received AS, 50% were sub-optimal, with 78/287 (27%) receiving the first dose less than 6 hours prior to delivery, and 67/287 (23%) receiving the first dose more than 7 days before their delivery. Twenty-four of the 287 (8%) received more than one course of AS. (Fig. 1)

The mean GA at delivery in the AS group was 35.6 weeks (SD 0.9) while the mean GA in the No AS group was 36.0 weeks (SD 0.8, p < 0.001). Among the group of mothers who received AS, 47 (16.3%) had some form of diabetes compared to 74 (34.4%) in the No AS group, (p < 0.001). A breakdown of diabetes classification for these mothers is included in Table 1. The incidence of PPROM was significantly lower in the AS group compared to the No AS group (32.6% vs. 35.1%, p < 0.005). The median time from admission to delivery was 19.4 hours (IQR of 6.9–48.1) in the AS group and 9.7 hours (IQR of 3.0–22.3) in the No AS group (p < 0.001). Table 1 summarizes the demographic characteristics for mothers in the AS and No AS groups.

### Infants

The mean birth weight among infants was 2522 g (SD 426 g) in the AS group and 2773 g (SD 457 g) in the No AS group (p < 0.001). This difference persisted after adjusting for GA as well as maternal characteristics including smoking status, pre-eclampsia, and any maternal diabetes. The mean Fenton Z score for birth weight in the AS group was −0.062 (SD 0.87), and those in the No AS group had a mean of 0.183 (SD 0.94, p < 0.05). There were no statistically significant differences between the two groups of infants classified as SGA or LGA.

We found no difference in the need for exogenous surfactant, need for mechanical ventilation, or in the average time spent on respiratory support between the two groups. The mean length of NICU stay was 4.8 days (SD 6.31) among infants who received AS and 3.4 days (SD 6.0, p < 0.05) among infants in the No AS group. Similarly, the mean length of stay in the hospital for infants who received AS was 7.1 days (SD 5.9), while it was 5.52 days (SD 5) for infants who did not receive AS (p = 0.001). In this dataset we found no difference in the incidence of hypoglycemia between infants who received AS and those who did not. The summary of infant characteristics and clinical outcomes are detailed in Table 2.

## Discussion

In the ALPS study, strict inclusion and exclusion criteria were utilized with the goal of identifying women who were likely to deliver a late preterm infant at least 24 hours after but within 7 days of betamethasone administration (but before term gestation). Despite this, our study shows administration was suboptimal in 50% of women who received AS, demonstrating the inherent difficulty of timing and predicting preterm delivery. Our study shows that mothers in the AS group delivered at an earlier mean GA compared to the no AS group. Considering that the median latency between the first dose to time of delivery was 0.78 days with IQ range of 0–6.36 days in the group who received AS, the lower mean GA at delivery within the AS group could reflect strict adherence to ALPS/SMFM criteria(1, 3), or a bias by providers towards not offering or administering AS the closer a pregnancy is to 37 weeks gestation.

In the study by McElwee, et al, the highest rate of inappropriate exposure was for PPROM without cervical change(10), which is in contrast to our data where the No AS group had a higher incidence of PPROM compared to those who received AS. Pregestational diabetes was an exclusion criteria for the ALPS study and SMFM recommendations, and our cohort expectedly showed a significantly larger proportion of diabetes in the No AS group compared to those given AS. The fact that some pregestational diabetics received AS however, indicates that the strict criteria is not being applied by all providers, or that AS was given under the protocol for those at risk of delivering prior to 34 weeks GA.

In contrast to the ALPS trial, the infants exposed to AS in our study were significantly smaller compared to the unexposed group, and the difference in birth weight remained significant even after adjusting for GA, maternal smoking status, maternal pre-eclampsia, and maternal diabetes (p < 0.001). However, we found no difference between the groups for those classified as SGA or LGA, although we were not powered for these outcomes.

We found no significant differences in short term neonatal respiratory outcomes, NICU admission, or overall hospital length of stay in the cohort exposed to AS even as we controlled for GA, but we are not powered to detect such difference in clinical outcomes. However, AS have been shown to increase passive respiratory compliance (Crs) and functional residual capacity (FRC) after birth in preterm infants exposed to AS within 7 days prior to delivery (12–15). We have demonstrated that in a single center cohort of 50 LPIs born between 34 0/7 to 34 6/7 weeks’ GA, a significant increase in Crs was noted for those treated with a single course of AS within 7 days of delivery versus matched infants who did not receive AS (1.33 vs 1.06 mL/cmH2O/kg, p = 0.018)(16). The infants treated with AS also had a higher functional residual capacity, and significantly less of the AS treated infants required continuous positive airway pressure treatment (16% vs 68%; p = 0.007)(16).

A specific harm associated with late preterm AS is neonatal hypoglycemia, which was identified as a significant outcome in the ALPS trial, occurring in 24% of those in the betamethasone arm (vs 15.0% in placebo with a relative risk of 1.60; 95% CI, 1.37 to 1.87; P < 0.001).(3) Although the definition remains controversial, transient and persistent neonatal hypoglycemia has been associated with negative long-term neurodevelopmental outcomes in preterm and term infants(17–23). Our study showed no difference in the incidence of hypoglycemia between the two groups using the same definition as the ALPS trial, which could be related to stricter adherence at our center to the exclusion criteria of any maternal diabetes. Only 4.5% of our AS group had pregestational diabetes, which was similar to the findings of the cohort by McElwee, et al, where only 3% of inappropriate AS exposures were in the setting of pregestational diabetes.(10) This is in contrast to the findings of Battarbee, et al, whose survey showed that greater than 50% of the providers reported administering betamethasone in the setting of poorly controlled diabetes or diabetes on insulin.(9)

Our study has several limitations. Due to the retrospective and non-randomized nature of our study, it was difficult to ascertain the precise indications or contraindications for AS administration unless it was documented by the provider. It was also difficult to determine other clinical variables that may have contributed to the decision for AS administration. Another limitation is that we did not examine term deliveries exposed to AS. In the ALPS study, 16.4% of patients went on to deliver at term, and this may be higher in a clinical setting leading to higher rates of non-optimal administration. This is concerning for a large body of obstetric and neonatal providers, given that long-term outcomes for AS exposure in LPIs remain unknown. Further, a recent population-based retrospective cohort study in Finland showed that AS exposure was significantly associated with higher risk of any mental and behavioral disorder in the entire cohort of term-born children (12.01% vs 6.45%; absolute difference 5.56% [95% CI, 5.04%–6.19%]; adjusted hazard ratio 1.33 [95%CI, 1.26–1.41])(7). In this study with 14,868 AS-exposed children, 6730 (45.27%) were born at term. These would be important populations and clinical outcomes for future prospective longitudinal studies to measure as risk and benefits are considered for the administration of corticosteroids during the period of late preterm gestation.

## Conclusion

The incidence of sub-optimal steroid administration continues to be high in our center despite close adherence to SMFM guidelines. AS administration did not improve neonatal outcomes, but we are underpowered for these clinical results. Prospective studies are needed to evaluate the impact of AS use in diabetics, to optimize the timing of AS dosing, and to evaluate the impact of AS on long term lung development in late preterm infants.

## Figures and Tables

**Table 1:** Demographics and clinical outcomes of pregnant women who delivered during the late preterm gestation between January 1, 2016 and December 31, 2020 at a single center

Maternal Characteristics			
	AS (n = 287)	No AS (n = 216)	P-Value
**Mean Age in Years (SD)**	31.4 (6.11)	31.7 (6.0)	0.666
**Mean gestational age (weeks) at delivery (SD)**	35.5 (0.90)	36.0 (0.80)	<0.001**
Hispanic vs. Not Hispanic			
**Non-Hispanic n (%)**	221 (77.0%)	147 (68.0%)	0.075 ^F^
**Hispanic n (%)**	56 (19.5%)	60 (27.8%)	
**Decline/Unknown n (%)**	10 (3.5%)	9 (4.2%)	
White vs. Not White			
**White n (%)**	231 (80.5%)	169 (78.2%)	0.058 ^F^
**Non-White n (%)**	44 (15.3%)	27 (12.5%)	
**Decline/Unknown n (%)**	12 (4.2%)	20 (9.3%)	
Delivery Method			
**Cesarean n (%)**	55 (25.0%)	33 (20.8%)	0.41 ^F^
**Vaginal n (%)**	165 (75.0%)	126 (79.2%)	
Diabetes Mellitus (DM)			
**Any DM n (%)**	47 (16.3%)	74 (34.4%)	<0.001**
**No DM n (%)**	240 (83.7%)	142 (65.5%)	<0.001**
**A1GDM n (%)**	16 (5.6%)	22 (10.2%)	
**A2GDM n (%)**	18 (6.25%)	27 (12.6%)	
**T1DM n (%)**	7 (2.4%)	8 (3.7%)	
**T2DM n (%)**	6 (2.1%)	17 (7.9%)	
Pregestational Diabetes			
**T1DM or T2DM n (%)**	13 (4.5%)	25 (11.6%)	0.004*
Hospital Course			
**Preterm Premature Rupture of Membranes n (%)**	93 (32.5%)	97 (45.0%)	0.005* ^F^
**Pre-Eclampsia/Gestational Hypertension n (%)**	103 (35.9%)	69 (31.9%)	0.756 ^F^
**Smoker n (%)**	29 (10.2%)	26 (12.2%)	0.093 ^F^
**Hours from Admission to Delivery, Median (IQR)**	19.4 (6.9–47.9)	9.7 (3.0–23.0)	< 0.001**
**Days from First Dose to Delivery, Median (IQR)**	0.8 (0–6.4)		

P values measured by T test for all numerical variables and Fishers exact for all categorical variables, noted with an ^F^.

* p < 0.05

** p<0.01

**Table 2:** Demographics and clinical outcomes of late preterm infants (LPIs) with and without exposure to antenatal steroids delivered at a single center between January 1, 2016 and December 31, 2020.

Infant Characteristics and Outcomes			
	AS (n = 287)	No AS (n = 216)	P value
**Female**	142	101	0.589 ^F^
**Male**	145	115	
**Birth weight**			
**Mean birth weight in grams (SD)**	2522 (426)	2773 (457)	<0.001* ^a^
**SGA (%)**	26 (9.1%)	10 (4.6%)	0.079 ^F^
**LGA (%)**	14 (4.9%)	21 (9.7%)	0.050 ^F^
**Mean Fenton Z-score for birth weight (SD)**	−0.062 (0.87)	0.183 (0.94)	0.003*
**Diagnoses and Hospital Course**			
**Median APGAR at 1 minute (IQR)**	7.35 (7–9)	7.24 (7–9)	0.55
**Median APGAR at 5 minutes (IQR)**	8.42 (8–9)	8.41 (8–9)	0.93
**LPIs who received exogenous surfactant**	3 (1.0%)	5 (2.3%)	0.29 ^F^
**LPIs who received mechanical ventilation**	7 (2.4%)	8 (3.7%)	0.38 ^F^
**Mean hours on respiratory support (SD)**	9.65 (25.8)	9.12 (27.0)	0.82
**Mean days in NICU (SD)**	4.79 (6.31)	3.37 (6.0)	0.01*^b^
**Mean days in hospital (SD)**	7.07 (5.9)	5.52 (5.0)	0.001*
**Hypoglycemia** ^c^	94 (43.7%)	121 (56.3%)	0.201 ^F^

P values measured by T test for all numerical variables and Fishers exact for all categorical variables (denoted by an ^F^).

* p < 0.05

SGA – small for gestational age, defined as birth weight < 10th percentile for gestational age

LGA – large for gestational age, defined as birth weight > 90th percentile for gestational age

a Adjusted for gestational age, smoking status, preeclampsia, and diabetes

b Adjusted for gestational age and birthweight

c Hypoglycemia defined by blood glucose < 40mg/dL within the first 24h of life.
